# Phase Ib study of berzosertib, carboplatin, gemcitabine, and pembrolizumab in patients with squamous nonsmall lung cancer (ETCTN 10313)

**DOI:** 10.1093/oncolo/oyaf373

**Published:** 2025-11-07

**Authors:** Liza C Villaruz, Benjamin Schluger, Hong Wang, James Ohr, Daniel Petro, Alexander D Fuld, Benjamin Herzberg, Richa Dawar, Jorge Nieva, Jimmy Ruiz, Susan M Christner, Julianne L Holleran, Christopher J Bakkenist, Steven Gore, Jan H Beumer

**Affiliations:** Cancer Therapeutics Program, UPMC Hillman Cancer Center, Pittsburgh, PA, 15232, United States; Division of Hematology/Oncology, Department of Medicine, School of Medicine, University of Pittsburgh, Pittsburgh, PA, 15232, United States; Cancer Therapeutics Program, UPMC Hillman Cancer Center, Pittsburgh, PA, 15232, United States; Division of Hematology/Oncology, Department of Medicine, School of Medicine, University of Pittsburgh, Pittsburgh, PA, 15232, United States; Cancer Therapeutics Program, UPMC Hillman Cancer Center, Pittsburgh, PA, 15232, United States; Department of Biostatistics, School of Public Health, University of Pittsburgh, Pittsburgh, PA, 15232, United States; Cancer Therapeutics Program, UPMC Hillman Cancer Center, Pittsburgh, PA, 15232, United States; Cancer Therapeutics Program, UPMC Hillman Cancer Center, Pittsburgh, PA, 15232, United States; Dartmouth Geisel School of Medicine, Hanover, NH, 03755, United States; Columbia University Irving Medical Center, New York, NY, 10032, United States; Sylvester Comprehensive Cancer Center, Miami, FL, 33136, United States; USC Norris Comprehensive Cancer Center, Los Angelos, CA, 90033, United States; Wake Forest Comprehensive Cancer Center, Winston-Salem, NC, 27103, United States; Cancer Therapeutics Program, UPMC Hillman Cancer Center, Pittsburgh, PA, 15232, United States; Cancer Therapeutics Program, UPMC Hillman Cancer Center, Pittsburgh, PA, 15232, United States; Department of Radiation Oncology, UPMC Hillman Cancer Center, School of Medicine, University of Pittsburgh, Pittsburgh, PA, 15232, United States; Investigational Drug Branch, Cancer Therapy Evaluation Program, Division of Cancer Treatment and Diagnosis, National Cancer Institute, Bethesda, MD, 20892, United States; The Sidney Kimmel Cancer Center at Johns Hopkins, Baltimore, MD, 21287, United States

**Keywords:** squamous NSCLC, ATR inhibition, chemotherapy, berzosertib

## Abstract

**Background:**

Berzosertib is a potent and selective inhibitor of ataxia telangiectasia Rad3-related and potentiates cisplatin and gemcitabine in lung xenografts. We hypothesized that berzosertib plus carboplatin, gemcitabine, and pembrolizumab is tolerable and active in newly diagnosed advanced squamous NSCLC.

**Methods:**

This phase Ib clinical trial studied berzosertib 135 mg/m^2^ IV Days 2 and 9 with carboplatin AUC 4-5 Day 1, gemcitabine 800 mg/m^2^ Days 1 and 8, and pembrolizumab 200 mg Day 1, of a 3-week cycle. Primary endpoint was the recommended phase II dose (RP2D). Antitumor activity and pharmacokinetics were secondary endpoints.

**Results:**

Twelve patients were enrolled and treated. Two of six patients at DL 1 (berzosertib 135 mg/m^2^, carboplatin area under the curve (AUC) 5, gemcitabine 800 mg/m^2^, and pembrolizumab 200 mg) experienced a dose-limiting toxicity: grade 5 gastric hemorrhage upon intractable nausea and vomiting; grade 3 neutropenia resulting in treatment delay. None of 6 patients experienced a DLT at DL-1 (berzosertib 135 mg/m^2^, carboplatin AUC 4, gemcitabine 800 mg/m^2^, and pembrolizumab 200 mg). The most common grade ≥3 TRAEs were leukopenia (75%), neutropenia (67%), anemia (58%), thrombocytopenia (33%), and lymphopenia (33%). Berzosertib and gemcitabine exposure did not correlate with the highest grade cycle 1 toxicity. Five of eleven evaluable patients (46%) experienced partial response.

**Conclusion:**

In newly diagnosed advanced squamous non-small cell lung cancer (NSCLC), berzosertib 135 mg/m^2^, carboplatin AUC 4, gemcitabine 800 mg/m^2^, and pembrolizumab 200 mg was the RP2D, which was both tolerable and associated with activity. This study did not proceed to phase II upon discontinuation of berzosertib development. Clinicaltrials.gov Identifier: NCT04216316.

Lessons Learned

- In patients with newly diagnosed advanced squamous NSCLC, the RP2D of berzosertib 135 mg/m^2^, carboplatin AUC 4, gemcitabine 800 mg/m^2^, and pembrolizumab 200 mg was associated with manageable side effects and antitumor activity.
- Higher doses of the chemotherapy backbone may be associated with prohibitive myelotoxicity.
- Although no further studies with berzosertib are planned in favor of orally bioavailable ataxia telangiectasia Rad3-related inhibitors, more studies are required to further define optimal dosing and schedule of ATR inhibitors with chemotherapy.

**Trial information** 

**Table:** 

Trial information	
**Disease**	Squamous NSCLC
**Stage of disease/treatment**	IV
**Prior therapy**	0
**Type of study**	Phase Ib
**Primary endpoints**	RP2D
**Secondary endpoints**	Progression free survival, overall survival, PK, safety and tolerability

**Drug information** 

**Table:** 

Drug information	
**Generic/working name**	Berzosertib
**Company name**	EMD Serono
**Drug type**	Targeted therapy
**Drug class**	ATR inhibitor
**Dose**	135 mg/m^2^
**Route**	IV
**Schedule of administration**	Day 2, 9, of a 21-day cycle

**Dose escalation table** 

**Table:** 

Dose escalation table						
Dose Level	Dose of drug				Number enrolled	Number evaluable for toxicity
	Berzosertib	Carbop­latin	Gemcitabine	Pembrolizumab		
	IV, D2, D9 (mg/m^2^)	IV, D1	IV, D1, D8 (mg/m^2^)	IV, D1 (mg)		
**1**	135	AUC 5	800	200	6	6
**−1**	135	AUC 4	800	200	6	6

**Table:** 

Patient characteristics	
**Number of patients, male**	5
**Number of patients, female**	7
**Age**	Median 65.5, range 50-80
**Race**	White 11 Unknown 1
**Performance status: ECOG 0**	5
**Performance status: ECOG 1**	7

**Table:** 

Primary assessment method	
**Title**	Progression free survival, overall survival, ORR
**Number of patients screened**	14
**Number of patients enrolled**	12
**Number of patients evaluable for toxicity**	12
**Number of patients evaluated for efficacy**	11
**Evaluation method**	RECIST 1.1
**Outcome notes** **In 12 patients, the median PFS was 9 months (95% CI: 3, 13) with median follow-up of 17 months. The median OS was 15 months (95% CI: 3, not reached) with a median follow-up of 17 months. Eleven of the 12 patients enrolled subsequently had at least one disease assessment after initiation of trial therapy in whom the ORR was 45.5% (5 of 11 patients with a partial response, 95% CI: 16.7%, 76.6%). The remaining 6 patients experienced stable disease (54.5%, 95 CI: 23.4%, 83.3%).**	

**Table:** 

Dose limiting toxicities		
Dose level	Adverse event(s), grade	Attribution
**1**	Gastric hemorrhage in the setting of intractable nausea and vomiting, Grade 5 Neutropenia resulting in significant delay of cycle 2 Day 1, Grade 3	Possible
**−1**	None	N/A

**Table:** 

Berzosertib geometric mean (SD) plasma pharmacokinetic parameters for Day 2 of cycle 1			
Dose level	Dose (mg/m^2^)	C_max_ (µg/L)	C_max_/Dose (µg/L/mg/m^2^)
**1**	**135 (*N* = 6)**	**700 (1.34)**	**5.18 (1.34)**
**−1**	**135 (*N* = 6)**	**640 (1.31)**	**4.74 (1.31)**
	**Total (*N* = 12)**	**669 (1.31)**	**4.99 (1.31)**

**Table:** 

Gemcitabine (dFdC) and metabolite (dFdU) geometric mean (SD) plasma pharmacokinetic parameters					
		dFdC	dFdU		dFdU/dFdC
Dose level	Dose (mg/m^2^)	C_max_ (mg/L)	C_max_ (mg/L)	AUC_0-24h_ (mg/L•h)	C_max_ MR
**1**	**800 (*N* = 6)**	9.29 (1.51)	23.9 (1.32)	264.9 (1.28)	2.57 (1.23)
**−1**	**800 (*N* = 6)**	11.2 (1.12)	22.1 (1.28)	242.9 (1.28)	1.98 (1.23)
	**Total (*N* = 12)**	10.2 (1.35)	23.0 (1.29)	253.7 (1.27)	2.26 (1.27)

Abbreviations: C_max_, maximum concentration; MR, metabolic ratio; SD, geometric standard deviation.

## Discussion

While significant therapeutic advances in squamous NSCLCs have been driven by the use of monoclonal antibodies targeting the PD1/PD-L1 pathway either alone or in combination with platinum-based doublet chemotherapy in the first-line treatment of advanced stages of disease, most patients still succumb to their metastatic disease.^1^ Ataxia telangiectasia mutated (ATM) and ataxia telangiectasia Rad3-related (ATR) protein kinases are the primary sensors of DNA damage and regulators of the DNA damage response (DDR).^2^ Inhibition of ATR has been shown to potentiate the cytotoxicity of cisplatin and gemcitabine in human NSCLC cell lines with intact ATM kinase signaling.^3^ In NSCLC xenograft models, the combination of ceralasertib (AZD6738) and cisplatin resulted in near-complete resolution of ATM-deficient NSCLC tumors, suggesting that ATM-deficient tumors are particularly sensitized to ATR inhibition in combination with platinum.^3^ Approximately 40% of NSCLCs have loss of ATM protein expression, identifying a significant proportion of lung cancer patients whose tumors may be exquisitely sensitized to ATR kinase inhibition.^4^

Berzosertib is a highly potent and selective ATP-competitive inhibitor of ATR.^5^ We hypothesized that berzosertib plus carboplatin, gemcitabine, and pembrolizumab is well tolerated and improves outcomes in newly diagnosed advanced squamous NSCLC. In this phase Ib, clinical trial, berzosertib 135 mg/m^2^ was administered IV on Days 2 and 9 with carboplatin AUC 4-5 on Day 1, gemcitabine 800 mg/m^2^ on Days 1 and 8, and pembrolizumab 200 mg/m^2^ on Day 1 of a 21-day cycle. The primary endpoint was determination of the recommended phase II dose (RP2D). Antitumor activity and pharmacokinetics were secondary endpoints.

The protocol was written to proceed to a randomized phase II clinical trial of carboplatin, gemcitabine, and pembrolizumab with and without berzosertib after the determination of the RP2D in phase Ib. The primary endpoint of the phase II was to estimate progression-free survival (PFS) in an all-comer squamous NSCLC population, with a key secondary endpoint to assess PFS in patients with ATM deficient squamous NSCLC. Unfortunately, after completion of phase Ib, the trial was terminated as no further clinical studies with berzosertib were planned in favor of orally bioavailable ATR inhibitors, such as tuvusertib.^6^

Twelve patients were enrolled and treated in phase Ib. Two of the six patients treated at DL 1 (berzosertib 135 mg/m^2^, carboplatin AUC 5, gemcitabine 800 mg/m^2^, and pembrolizumab 200 mg) experienced a dose-limiting toxicity: grade 5 gastric hemorrhage in the setting of intractable nausea and vomiting, and grade 3 neutropenia resulting in significant delay of cycle 2 Day 1. The former patient passed away at home after having received cycle 1 Day 9 of therapy and causality was based on clinical history. While this event is not typical of the components of this regimen, we conservatively determined the event to be at least possible related to the platinum backbone of therapy and deemed this a DLT. None of the 6 patients treated at DL-1 (berzosertib 135 mg/m^2^, carboplatin AUC 4, gemcitabine 800 mg/m^2^, and pembrolizumab 200 mg) experienced dose limiting toxicity (DLT). Most common treatment-related adverse events (TRAEs) were anemia (83%), neutropenia (83%), leukopenia (83%), thrombocytopenia (58%), ALT increase (50%), lymphopenia (50%), and nausea (50%). The most common grade ≥3 TRAEs were leukopenia (75%), neutropenia (67%), anemia (58%), thrombocytopenia (33%), and lymphopenia (33%).

Due to the limited sampling, the most reliable PK parameter assessed was C_max_. Observed values for berzosertib were comparable to previous reports.^7^^,^^8^ The reported human equivalent p-Chk1 IC_50_ (half the maximum inhibitory concentration of Chk1, a primary downstream target of ATR) of approximately 110 ng/mL was exceeded in all patients for several hours. The pharmacokinetics of gemcitabine and its metabolite dFdU were also in line with previous reports.^9^ Drug exposures for either drug did not appear to correlate with the highest grade toxicity in this small dataset.

In 12 patients, the median PFS was 9 months (95% CI: 3, 13) with median follow-up of 17 months. The median OS was 15 months (95% CI: 3, not reached) with a median follow-up of 17 months. Eleven of the 12 patients enrolled subsequently had at least 1 disease assessment after initiation of trial therapy in whom the ORR was 45.5% (5 of 11 patients with a partial response, 95% CI: 16.7%, 76.6%). The remaining 6 patients experienced stable disease (54.5%, 95 CI: 23.4%, 83.3%). While no conclusions can be made with regard to the efficacy of this regimen in patients with newly diagnosed advanced squamous NSCLC, these response and survival data are in line with the prior phase III studies that established the role of chemoimmunotherapy in this patient population.^10^ As this clinical trial did not proceed with phase II, we were unable to complete meaningful correlative analyses of the impact of ATM expression on efficacy.

In 23 patients treated on a prior phase I clinical trial evaluating doses of berzosertib 90-240 mg/m^2^ and carboplatin AUC 4-5, there were no events of grade ≥3 nausea and vomiting, suggesting the DLT of grade 5 gastric hemorrhage in the setting of intractable nausea and vomiting is not typical of this regimen.^5^ The current study demonstrated that the combination of berzosertib with carboplatin and gemcitabine was associated with significant myelosuppression with 67% of patients experiencing grade ≥3 neutropenia. In the prior phase I clinical trial evaluating berzosertib and carboplatin, 22% of patients experienced grade ≥3 neutropenia.^5^ Notably, rates of hematologic toxicities in the current study were also higher than what was seen with chemoimmunotherapy in a similar population of patients.^10^

Myelosuppression to varying degrees has been seen throughout the development of ATRi chemotherapy combinations. Notably, a phase II clinical trial comparing gemcitabine and cisplatin plus berzosertib with gemcitabine and cisplatin alone demonstrated inferior survival with the addition of berzosertib in patients with advanced urothelial carcinoma.^11^ Higher rates of grade 3 and 4 thrombocytopenia and neutropenia and more dose reductions in the berzosertib-containing arm, resulting in a statistically significant lower exposure to cisplatin, may explain the inferior outcomes in the berzosertib-containing arm. We were not able to proceed with the phase II portion of our study, so it is unclear how these results would have translated to the first-line treatment of advanced NSCLC.

In conclusion, berzosertib 135 mg/m^2^, carboplatin AUC 4, gemcitabine 800 mg/m^2^, and pembrolizumab 200 mg were the RP2D in patients with newly diagnosed advanced squamous NSCLC and were associated with manageable side effects and antitumor activity. While no further studies with IV berzosertib are planned, in favor of orally bioavailable ATR inhibitors, our study suggests ATR inhibition with induction chemoimmunotherapy in the first-line treatment of patients with advanced squamous NSCLC may be a viable therapeutic strategy, though further studies would be needed to delineate if efficacy of combining ATR inhibitors with chemotherapy may ultimately be limited by myelosuppression.

## Data Availability

The data generated in this study are publicly available in ClinicalTrials.gov (NCT02595931).
